# Key process features of personalized diet counselling in metabolic syndrome: secondary analysis of feasibility study in primary care

**DOI:** 10.1186/s40795-022-00540-9

**Published:** 2022-05-09

**Authors:** Paula Brauer, Dawna Royall, Airu Li, Ariellia Rodrigues, Jennifer Green, Sharon Macklin, Alison Craig, Miranda Chan, Jennifer Pasanen, Lucie Brunelle, Rupinder Dhaliwal, Doug Klein, Angelo Tremblay, Caroline Rheaume, David M. Mutch, Khursheed Jeejeebhoy

**Affiliations:** 1 Department of Family Relations & Applied Nutrition, University of Guelph, 50 Stone Road East, Guelph, ON N1G 2W1 Canada; 2 Current address: Trillium Health Partners, Mississauga, ON Canada; 3 Edmonton Oliver Primary Care Network, Edmonton, AB Canada; 4 Department of Kinesiology, Laval University, Québec, QC Canada; 5 Metabolic Syndrome Canada, Kingston, ON Canada; 6 Current address: Canadian Nutrition Society, Kemptville, ON Canada; 7 Department of Family Medicine, University of Alberta, Edmonton, AB Canada; 8 Department of Family Medicine and Emergency Medicine, Laval University, Québec, QC Canada; 9 Department of Human Health & Nutritional Sciences, University of Guelph, Guelph, ON Canada; 10 Department of Medicine, University of Toronto, Toronto, ON Canada

**Keywords:** Implementation, Process, Health behaviour change, Behaviour change techniques, Nutrition care process, Personalized diet counselling, Cardiometabolic conditions, Metabolic syndrome

## Abstract

**Background:**

Personalized diet counselling, as part of lifestyle change programs for cardiometabolic risk conditions (combinations of prediabetes or type 2 diabetes, hypertension, dyslipidemia and high waist circumference) has been shown to reduce progression to type 2 diabetes overall. To identify key process of care measures that could be linked to changes in diet, we undertook a secondary analysis of a Canadian pre-post study of lifestyle treatment of metabolic syndrome (MetS). Diet counselling process measures were documented and association with diet quality changes after 3 months were assessed. Results of the primary study showed 19% reversal of MetS after 1 year.

**Methods:**

Registered dietitians (RDs) reported on contact time, specific food behaviour goals (FBG), behaviour change techniques (BCT; adapted from the Michie CALO-RE taxonomy) and teaching resources at each contact. Diet quality was measured by 2005 Canadian Healthy Eating Index (HEI-C) and assessed for possible associations with individual BCT and FBG.

**Results:**

Food behaviour goals associated with improved HEI-C at 3 months were: poultry more than red meat, increased plant protein, increased fish, increased olive oil, increased fruits and vegetables, eating breakfast, increased milk and alternatives, healthier fats, healthier snacks and increased nuts, with an adverse association noted for more use (> 2 times/ 3 months) of the balanced meal concept (F test; *p* < 0.001). Of 16 BCT, goal setting accounted for 15% of all BCT recorded, yet more goal setting (> 3 times/3 months) was associated with poorer HEI-C at 3 months (F test; *p* = 0.007). Only self-monitoring, feedback on performance and focus on past success were associated with improved HEI-C.

**Conclusions:**

These results identify key aspects of process that impact diet quality. Documentation of both FBG and BCT is highly relevant in diet counselling and a summary diet quality score is a promising target for assessing short-term counselling success.

**Supplementary Information:**

The online version contains supplementary material available at 10.1186/s40795-022-00540-9.

## Background

Cardiometabolic risk (CMR) conditions and diseases are a major and growing health burden in many countries, as obesity continues to increase worldwide [1]. As body weight increases overall in the population, a subset tend to accumulate excess visceral abdominal and ectopic fat which has been shown through body composition studies to be more strongly associated with cardiovascular disease (CVD) risk than excess body weight per se [2]. Adverse metabolic effects become more prominent in middle age and are variously defined in health systems as specific conditions, such as prediabetes, type 2 diabetes, or hypertension, or risk scores such as the Framingham 10-year CVD score [3]. Metabolic syndrome (MetS) is characterized by three or more indicators including higher waist circumference, higher blood pressure, dyslipidemia characterized by low high-density lipoprotein and elevated triglyceride levels, and elevated glucose levels [4]. The various terms describe overlapping populations [5], and different combinations of risk factors in populations may differentially affect CVD risk [6, 7].

World-wide prevalence of some risk factors like hypertension, obesity and type 2 diabetes are well documented [8], while pre-clinical conditions like prediabetes [9] or combinations like MetS have been less often assessed in national surveys [10]. Canadian data from the Canadian Health Measures Survey confirm high prevalence of CMR conditions. Prediabetes affected 12.4% of Canadian adults 20–79 years from the 2007–2011 surveys [11], while 21% had MetS in the 2012–2013 survey [12]. Among Canadian adults with MetS, 92% had high waist circumference, 74% had high triglycerides, 70% low HLD-C, 61% elevated glucose and 58% high blood pressure. People with MetS have 1.5 to 2 times the CVD risk of those without the syndrome, according to the Framingham score [13]. Ongoing costs of CMR are substantial, as confirmed in a 2016 US study of the Medical Expenditure Panel Survey. Among those with 3 or 4 risk factors compared to those with none of the CMR conditions, health care utilization was 50% higher, days missed from work 75% higher and yearly heath care costs more than twice as high [14]. In addition, recent experience with COVID-19 has confirmed increased risk of severe disease in the presence of these common conditions, although estimates vary [15, 16].

The overall benefits of personalized diet counselling, also called medical nutrition therapy, in secondary prevention of CMR conditions is well established, yet it has been challenging to link key features of such counselling to intermediate outputs, such as changes in food intake. Several large clinical trials of health behaviour change have now demonstrated reductions in CVD mortality and diabetes incidence, namely the PREDIMED study [17, 18] and the Diabetes Prevention Program [19–21] and subsequent studies [22, 23]. The United Kingdom has been implementing a national diabetes prevention program and publishing experience to date [24, 25]. With respect to the dietary aspects of these studies, the main foci to date have been on the dietary goals and achieved changes in clinical indicators; with promotion of a Mediterranean diet in PREDIMED or general weight loss in most other studies [26, 27]. As evidence on the key metabolic aberrations continues to accumulate, undoubtedly the targets for lifestyle interventions in CMR conditions will continue to evolve [2]. For example, interventions to reduce overall body weight have favoured diet approaches, while greater reduction in visceral abdominal fat is achieved by exercise in clinical studies to date [28]. Change in diet quality, as achieved in PREDIMED, also appears to be helpful. Whatever the focus, linking features of the counselling process itself to changes in dietary intake has received limited attention, with a significant review last done by Desroches et al. in 2013 [29].

Translating clinical trial results to health care practice settings, the so-called efficacy-effectiveness gap, has several challenges. Challenges include: 1) diversity of patient interest, capability and skills compared to clinical trial volunteers (Hawthorne effect), 2) efficacy of different diets [30], 3) measurement challenges in assessing diet and diet change in practice, 4) the diversity of motivations, values, psychological and physiological factors that impact eating patterns, 5) measurement issues in identifying the key aspects of the counselling process, and 6) linking the care process to key changes in food consumption or clinical measures. In CMR conditions, patients may focus on weight loss as a main outcome, yet there is strong evidence that most will not achieve much weight loss, whereas changes in diet quality may also have benefits [31].

Some of the methodological challenges of health behaviour change are very similar across different health issues. Experts have long argued for improved description of interventions with regard to design, interventions, setting, and population group to potentially identify key factors important to successful behaviour change [32–35]. While some progress has been made, measurement of care process with linkage to dietary change has been slow in diet counselling studies [36, 37]. We therefore undertook a secondary analysis of a successful feasibility study of lifestyle change in team-based primary care in Canada that had demonstrated 19% reversal of MetS over 1 year, associated with 2.5 kg mean maximum likelihood weight loss [38]. We first documented process over the whole 12 months, and then focused on analysis of the first 3 months of weekly counselling when most dietary change occurred [39]. The goal of this work was to assess whether and to what degree personalized diet counselling could be linked to changes in diet quality, as measured by 2005 Canadian Healthy Eating Index (HEI-C) [40].

## Methods

### Primary study design and results

Our research group implemented an intensive, structured lifestyle intervention program (Canadian Health Advanced by Nutrition & Graded Exercise, CHANGE project) led by family physicians (FP) that involved Registered Dietitians (RDs) and kinesiologists in primary care settings. Patients with MetS were enrolled from three participating clinics located in Edmonton, Toronto, and Quebec City. We hypothesized the intervention would be feasible in the Canadian primary care system and would result in reversal or reduction in the components of MetS at 12 months. The program was flexible, given the variety of ways that primary care is organized in Canada [41]. Recruitment began in Oct 2012 and was completed in Dec 2014. Details on overall study methods are provided elsewhere [38, 42]. Briefly, each patient was assessed by their FP and referred to the RD and kinesiologist for individual assessment and lifestyle intervention. All patients were followed by the RD and kinesiologist weekly for the first 3 months, then monthly for the next 9 months. The FP followed each patient at 3, 6, 9 and 12 months for a review of blood work, assessment of progress and ongoing encouragement. Ethics approvals for the study were obtained from Health Research Ethics Board- Biomedical (University of Alberta), Comité d’éthique de la recherche des Centres de santé et de services sociaux de la Vieille-Capitale (Laval University), University of Guelph Research Ethics Board, and Institutional Review Board Services, a Chesapeake IRB Company (Aurora, Ont.).

A total of 293 adult patients with MetS were recruited (52% female, mean ± SD age 59.1 ± 9.7 years) presenting primarily with elevated waist circumference (95%), elevated blood pressure (87%) and elevated blood glucose (82%). At 12 months, 19% of patients (95% CI, 14 to 24%) showed reversal of MetS [38]. Papers addressing the nutrient and food group changes [39], exercise changes [43] and patient experience [44] have been published. Study completers compared to non-completers did not differ by gender, but were older, at higher CVD risk and had lower BMI [38, 39].

### Dietary intervention

Fourteen RDs employed by the clinics were involved in counselling over the course of the study. While guided by a care map that focused mainly on possible food intake changes [45], they practiced according to professional norms current in 2011 and local organization practices. Such counselling is a personalized, systematic process that includes assessment, planning or diagnosis, implementation, and evaluation [46–48]. RDs typically use motivational interviewing, mutually setting achievable goals within defined time periods, involving follow-up visits, self-monitoring, and engaging social support [49].

Weight loss as the main focus in diet counselling practice has been controversial [50, 51], as weight loss is highly variable even in well-controlled studies [52], and weight regain almost universal [53]. The care map developed for this study included a weight loss focus where feasible, as decided by the counsellor and client [45], but also promoted qualitative diet changes, consistent with principles of several healthy dietary patterns, including the 2007 Canada’s Food Guide [54] and principles of a Mediterranean style diet [55].

Each RD received orientation to the project methods either in-person or via a 60–90 minute teleconference call, using a PowerPoint slide deck to anchor discussion. Dietitians also received several newly developed patient handouts on lifestyle treatment of MetS and an example joint goal setting guide [56] in addition to a 60-page counselling support document with links to diverse patient teaching resources such as label reading, recipes, etc. This resource was developed by two MSc-trained practicing RDs and organized by the care map topics [45]. All the resources were available via cloud-based folders (Dropbox, TM) for the RDs to access online, in numbered categories for the RDs to document resources used. Ongoing support to RDs was provided by a listserv and periodic teleconferences held throughout the study to encourage sharing of experience and resources.

### Development and review of process indicators

Possible self-report process measures were identified from several sources. Work from the Cochrane Effective Practice and Organization of Care (EPOC) Group [57, 58], along with review of relevant diet systematic reviews [59]. Common process metrics have included overall intervention duration, number of contacts, setting, theoretical background, qualifications/disciplinary background of interventionists, group vs individual approaches, and technology used. Within dietetics, the use of the Nutrition Care Process (NCP) has been ongoing since the 1970s [60], with the US Academy of Nutrition and Dietetics disseminating a standardized set of terms to describe the diet counselling process beginning in 2002 [61, 62]. While mainly used in the past to document care for all types of nutrition issues in medical records, studies are underway to assess use of NCP terminology against clinical outcomes [48]. While the NCP was too generic for our purposes, we used three of the five NCP intervention categories: 1) food and nutrient delivery; 2) nutrition education; and 3) nutrition counselling to inform our thinking on possible measures.

### Encounter time and channels

Contact in primary care was mainly in-person when the CHANGE project started, but group programs, email and telephone counselling were also available [63]. Dietitians therefore were asked to report each encounter date, setting (individual or group), presence of a support person, the delivery method (face-to-face, phone, or email), and the contact time in minutes.

### Behaviour change techniques (initial development, changes in pilot testing)

In the UK, Michie and colleagues have published three iterations of their behaviour change technique (BCT) taxonomies [35, 64, 65]. BCTs have been defined as the smallest identifiable components that in themselves have the potential to change behaviour [66]. The first 26-item version was expanded to a 40-item version in 2011 (Coventry Aberdeen and London—Refined version or CALO-RE) [64]. The CALO-RE taxonomy had only been used in paper review of research studies at the outset of the CHANGE project [64], and was therefore reviewed by the research team for applicability to ongoing RD recording of BCT in practice.

First, the research group removed BCTs not used in dietetics from the CALO-RE list (e.g. fear). Next, a video of a mock 45-minute initial counselling session for CVD risk (unscripted, RD and professional actor) was created (available from corresponding author). The team of RDs independently reviewed the video to identify core BCTs, using the definitions given in the original CALO-RE list (See Additional file 1 for the list of BCTs used). Reliability assessment revealed the RDs agreed 100% on the top four major BCTs observed, but not more. Several teleconferences were then held with study RDs to achieve consensus on the 16 BCTs to include, plus a write-in option, to achieve a reduced list that was feasible to complete. Dietitians used as many BCTs as they felt necessary during counselling sessions but were asked to record a maximum of the four most important BCTs per session. All RDs were trained on recording BCTs using the same video.

### Skill building activities

RDs were asked to write in any skill building activities recommended, such as participating in cooking classes, grocery store tours, learning portion sizes, or recording food intake etc. Recording of food intake was a key activity throughout the counselling process, so the BCT for self-monitoring was recorded when food records were being kept. Other skill activities were recorded separately and not coded as BCTs.

### Food behaviour goals list development

The food behaviour goals (FBG) list was initially developed by the research team, based on research evidence [45]. Components from the Mediterranean diet were incorporated, including *Increase olive oil*, *Increase fish*, *Poultry more often than red meat* and *Wine if consuming alcohol. C*omponents were also added from review by the RDs, including *Balanced meals*, *Regular meal pattern*, *Increase milk and alternatives*, *Decrease alcohol* and *Mindful eating approaches*, to make a final list of 24 FBGs and “other”, a write-in option. There was no limitation on the number of FBG that could be recorded. See Additional file 2 for the complete list of FBG used in counselling sessions. Initially it was envisaged that food goals would be set, worked on over time and achieved, as conceptualized in the older adherence and action planning literature [29, 67, 68]. This idea was abandoned based on feedback from the RDs that participants often cycled back to goals over time, and they were unable to document when changes were achieved (Stevens, undergraduate thesis results). Other FBG goals were diverse and not analysed further.

### Patient resources – development and capture

RDs often provided take-home handouts and pamphlets to clients to reinforce skill development, knowledge and self-monitoring. There were 56 categories of resources provided to all RDs, with several identified in each category. Efforts were made to include both basic and advanced resources. RDs could document usage by citing the resource category number. Three categories of resources could be recorded at each encounter on the data collection forms (supplement to primary paper [38]).

### Diet quality assessment – HEI-C 2005

Detailed dietary assessment was performed at baseline, week 12 and month 12, as previously described [39]. Briefly, patients reported on food intake by multi-pass 24-hour recall conducted in-person with the RD, twice at each time point, and the RDs completed a food frequency questionnaire with each patient for calculation of the HEI-C (range 0 to 100) [40]. See Additional file 3 for the scoring criteria used to calculate HEI-C.

### Data collection

A draft data collection form was reviewed for face validity and feasibility by the research team and the initial RD group, using cognitive interviewing and a focus group, prior to addition to the online data capture system. The nutrition process data were entered mainly as fill-in data (e.g., contact time, resources) or “check all that apply” dropdown menus (BCT and FBG) into a secure online data capture system (REDCap: http://www.project-redcap.org/) by the RDs themselves. The data capture system included data restrictions and real-time data integrity checks. Dietary intake data were analyzed at the University of Guelph and summary measures entered in the REDCap system. Detailed dietary data was merged with demographic and clinical data at each time point using SPSS Statistics 28.

### Analysis

Process and dietary data were first descriptively analysed at baseline, 3 months and 12 months for total mentions and mentions/patient/contact. Detailed analysis of dietary data indicated that most changes occurred by the 3-month assessment coinciding with the study design of weekly and monthly contacts [39]. RDs varied in their completion of the process measures; so more detailed analyses of text entries on other FBG, resources and skills were completed. It was noted that documentation of self-monitoring as a skill building activity overlapped with self-monitoring as a BCT, accounting for 70% of all skills documentation. Since self-monitoring is a critical aspect of health behaviour change counselling, the data was checked to ensure that any mention of self-monitoring as a skill was also documented as a BCT.

As we were interested in identifying if and which BCT and FBG were associated with changes in diet quality, only participants with complete dietary data to 3 months were included in the association analysis. Mentions of BCT and FBG were tabulated, and categorized, most commonly as no mention or mentioned (i.e., 0,1), or in approximately equal categories. To assess if use of each technique was associated with 3-month total HEI-C score, one-way ANOVA, adjusted for baseline HEI-C score was done first. Levene’s test for equality of error variance of the dependent variable was checked. Associations were adjusted for the following possible confounders and effect modifiers: sex, age, presence of a spouse (y/n), baseline BMI, baseline percentile VO_2_ max (a measure of fitness) and baseline PROCAM score (measure of CVD risk) [69]. The Bonferroni test was used for post-hoc comparisons to adjust for multiple comparisons within each BCT or FBG. The other groups of FBG and BCTs were excluded from the association analysis. Associations among strategies were ignored for this basic analysis.

## Results

Of the 293 participants enrolled in the study, 255 (87%) had complete data (HEI-C) at 3 months and 206 (70%) completed the 12-month study. Detailed results on HEI-C have been published [39]. HEI-C ± SD improved from 58 ± 15 to 69 ± 12 at 3 months and was maintained at 12 months, on a 0–100 scale. Overall change in HEI-C ± SEM was + 10.9 ± 1.03 points from baseline to 3 months (*p* < 0.001), and − 1.2 ± 0.98 from 4 to 12 months (not significant) [39].

### Encounter time and contacts

Since detailed diet assessment (two 24-hour recalls and HEI-C scoring) were conducted at baseline, 3 month and 12-month meetings, mean contact at these time points was longer (50 minutes; range 20–100 minutes) and decreased to ~ 30 minutes for each weekly visit in the first 12 weeks and monthly visits thereafter. In the first 3 months, most weekly visits were face-to-face (87%) with only a small number of encounters occurring by phone (11%) and email (2%). During the weekly visits in the first 3 months, a family member was present at 6.2% of the meetings.

### Behaviour change techniques

**Fig. 1:**
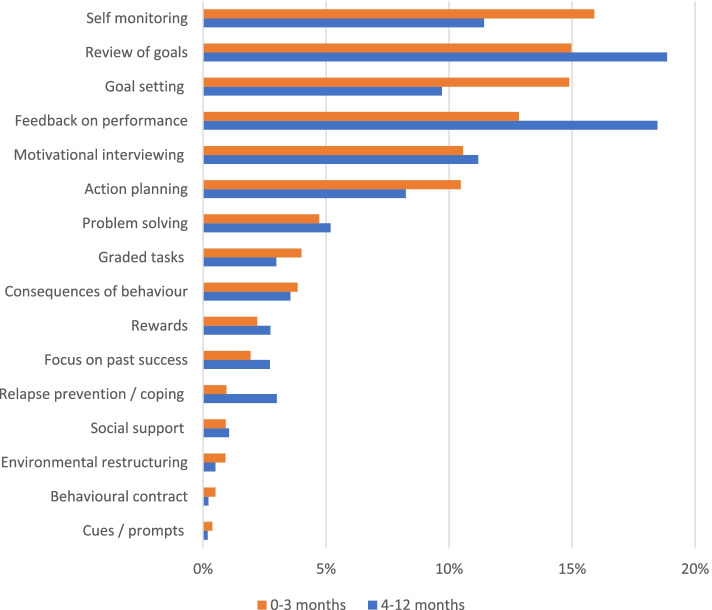
Frequency of each behaviour change technique (BCT) used by the dietitians. Results are shown during 0 to 3 months and 4 to12 months, displayed as percentage of all BCT during each period, ranked by percentage calculated over the first three months. Over the first 3 months, there were 7049 mentions of BCTs with an average of 2.0 BCT per person/contact and 4634 mentions over 4–12 months or 2.2 BCT per person/contact

As shown in Fig. 1, the top six BCT each accounted for at least 10% of mentions in either baseline to 3-month period or the 4 to12 month period or both. These were: *Self monitoring*, *Review of goals, Goal setting, Feedback on performance, Motivational interviewing* and *Action planning,* all of which are interconnected. Interestingly, several other important techniques, such as *Relapse prevention* and *Social support* were rarely documented.

### Food behaviour goals and resources

**Fig. 2:**
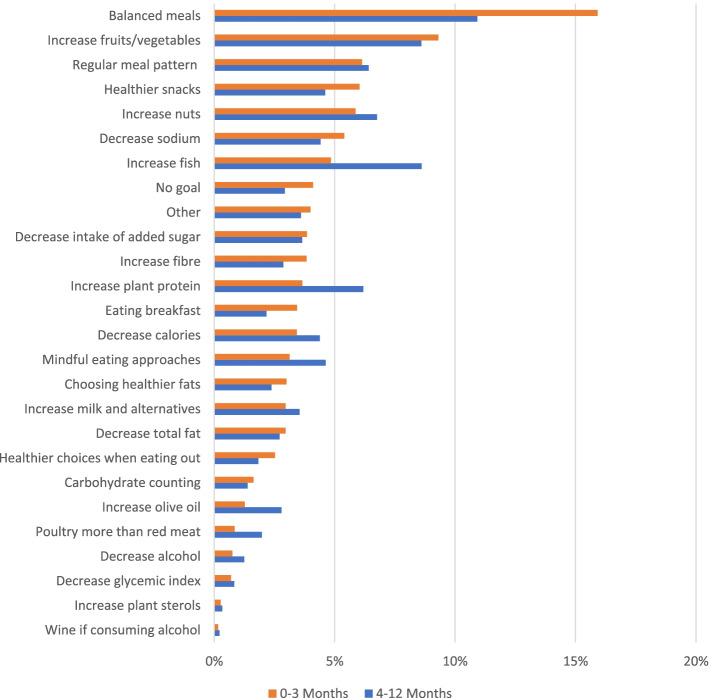
Frequency of each food behaviour goal (FBG) being selected by the dietitians. Results are shown during 0 to 3 months and 4 to 12 months, displayed as percentage of all FBG used during each period, ranked by percentage calculated over the first three months. Over the first three months, there were 8283 mentions of FBG, with an average of 2.3 goals per person/contact, and 8293 mentions over 4–12 months, or 2.7 goals per person/contact

As shown in Fig. 2, *Balanced meals* and *Increase fruits/vegetables* were the most frequently used FBG, especially *Balanced meals* which was used approximately 15% of total times when RDs chose a FBG in the first 3 months.

**Fig. 3:**
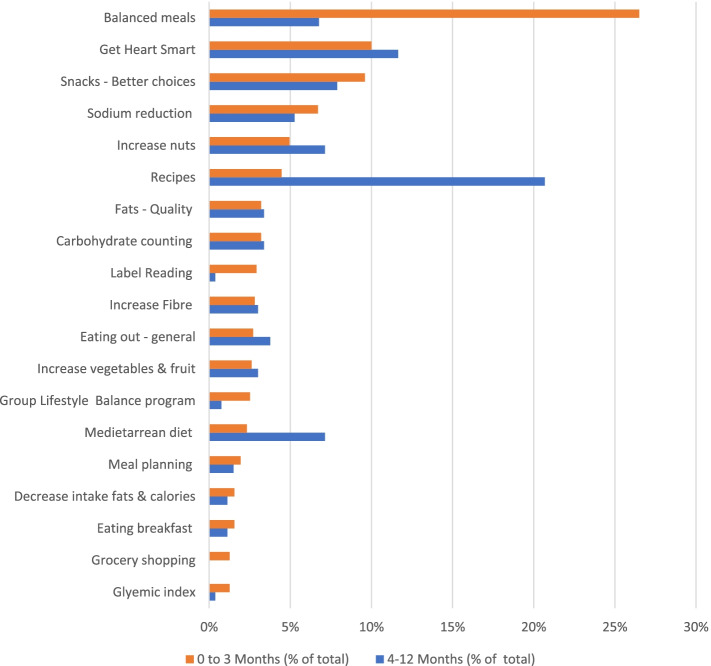
Frequency of each resource being used by the dietitians. Results are shown during 0 to 3 months and 4 to12 months, displayed as percentage of all resources used during each period, ranked by percentage calculated over the first three months. Over the first 3 months, there were 1030 resources used and 266 resources used over 4–12 months

In line with the high frequency of mentions of *Balanced meals* as a FBG, there was substantial use of Balanced meal handouts in the first 3 months, which included Canada’s Food Guide and balanced plate model resources (see Fig. 3). The second most used resource was ‘Get Heart Smart’ a compendium of one-page handouts put together by a RD group in Niagara region of Ontario [70]. French, English and few Russian resources were used, but use by language was not recorded.

### Skill-building

Skill-building activities were input as text in the data collection form and there was overlap with both BCT and FBG. Self-monitoring of food and/or glucose were by far the most often mentioned activity under skill building; 69% of all mentions baseline-3 months and 72% at 4–12 months, or 0.4 mentions/person/contact baseline-3 months and 0.3 mentions/person/contact, respectively. The number of mentions of skill building activities over 12 months, not already addressed as a FBG or BCT included: label reading (*n* = 79), meal preparation techniques (*n* = 58), grocery store tour (*n* = 9), grocery shopping techniques (*n* = 8), stress management (*n* = 5), cooking class (*n* = 4), program on emotional eating (*n* = 5), smoking cessation (*n* = 3), time management (*n* = 1), sleep management (*n* = 1); these mentions could have been to other local programs.

### Associations with change in diet quality at 3 months

Review of associations with HEI-C at 3 months revealed that more frequent mentions (> 3) of *Goal setting* was associated with a lower HEI-C at 3-months, compared to no mention or 1–3 mentions (Table 1). More *Self-monitoring* was associated with > 6 point higher HEI-C compared to 1–4 mentions, with no mentions having an intermediate HEI-C value. More *Feedback on performance* and no mention were each associated with lower HEI-C at 3-months, compared to 1–3 mentions. *Focus on past success* was associated with a significant increase in HEI-C. None of the other 12 BCTs were associated with diet quality at 3-months. Among the six BCTs most often mentioned, *Goal setting*, *Self-monitoring* and *Feedback on performance* were statistically significantly associated with 3-month HEI-C, while *Review of goals* was possibly associated (F test *p* = 0.06), but *Motivational interviewing* and *Action planning* were not associated with HEI-C at 3-months.

**Table 1:** Analysis of Variance for univariate and adjusted associations of each behaviour change technique (BCT) with 3-month HEI-C, adjusted for baseline HEI-C

BCT	Categories (all 0,1 except as indicated)	Univariate F ***P*** value^**a**^	Adjusted F ***P*** value^**b**^	Estimated Marginal Mean HEI-C at 3 Months – adjusted analysis
**Decreased HEI-C**				
Goal setting	0, 1–3, >3^c^	**0.007**	**0.007**	More than 3 mentions associated with lowest HEI score (66 points), none and 1–3 mentions had higher HEI (71 points).
**Increased HEI-C**				
Self-monitoring	0, 1–4, >4^d^	**0.000**	**< 0.001**	More than 4 mentions associated with highest HEI score (73 points), no mentions was intermediate (68 points) and 1–4 mentions had the lowest score (65 points)
Focus on past success		**0.009**	**0.018**	Mention associated with higher HEI score (71 points) compared to no mentions (67 points)
Feedback on performance	0, 1–3, >3^e^	**0.037**	**0.026**	More than 3 mentions and no mentions associated with lowest HEI score (67 points), 1–3 mentions had higher HEI (71 points).
**No difference**				
Review of goals	0, 1–4, >4^f^	0.061	0.064	
Consequences		0.078	0.046	
Environment re-structuring		0.096	0.054	
Social support		0.162	0.264	
Graded tasks		0.182^a^	0.164	
Action planning	0, 1–3, >3^g^	0.260	0.230	
Rewards		0.294	0.344	
Motivational Interviewing	0, 1–3, >3^h^	0.598	0.584	
Cues/prompts		0.639^a^	0.634	
Relapse prevention/coping		0.713^a^	0.762	
Behaviour contract		0.764	0.748	
Problem solving		0.798	0.919	

^a^ Levene’s test of equality of error variance of the dependent variable was *p* < 0.05 for the BCTs with superscript and otherwise *p* > 0.05

^b^Adjusted for sex, age, BMI, Baseline percentile VO_2_ max, baseline PROCAM score, spouse yn

^c^ Goal setting recoded as: 0 = not mentioned (*n* = 29); 1 = mentioned 1–3 times in 3 months (*n* = 111); 2 = mentioned more than three times (*n* = 115)

^d^ Self-monitoring recoded as: 0 = not mentioned (*n* = 83); 1 = mentioned 1–4 times in 3 months (*n* = 77); 2 = mentioned more than four times (*n* = 95)

^e^Feedback on performance recoded as: 0 = not mentioned (*n* = 50); 1 = mentioned 1–3 times in 3 months (*n* = 100); 2 = mentioned more than three times (*n* = 105)

^f^ Review of Goals recoded as: 0 = not mentioned (*n* = 48); 1 = mentioned 1–4 times in 3 months (*n* = 102); 2 = mentioned more than four times (n = 105)

^g^ Action Planning recoded as: 0 = not mentioned (*n* = 76); 1 = mentioned 1–2 times in 3 months (*n* = 71); 2 = mentioned more than two times (*n* = 108)

^h^ Motivational interviewing recoded as: 0 = not mentioned (*n* = 96); 1 = mentioned 1–2 times in 3 months (*n* = 65); 2 = mentioned more than two times (*n* = 94)

Table 2 shows the associations between specific FBG and HEI-C at 3-months, ordered from largest to smallest association. More mentions of *Balanced meals* (more than 2 times compared to fewer or no mentions was associated with lower 3-month HEI-C (F test *p* < 0.001). Mention of *Decrease alcohol* was associated with 6-point lower HEI-C values at 3 months (F test *p* = 0.03). FBG associated with > 6 point higher HEI-C at 3 months were *Poultry more than red meat*, *Increase plant protein, Increase fish and Eating breakfast*. The next group of six FBG all achieved statistically significant but smaller improvements in 3-month HEI-C. There was also nine FBG that were not associated with HEI-C at 3-months.

**Table 2:** Analysis of variance for univariate and adjusted associations of each food behaviour goal (FBG) with 3-month HEI-C, adjusted for baseline HEI-C

FBG variable name	Categories (all 0,1 except as indicated)	Univariate F ***p*** value^**a**^	Adjusted F ***p*** value^**b**^	Estimated Marginal Mean HEI-C at 3 Months – adjusted analysis
**Decreased HEI-C**				
Balanced meals	**0, 1–2,>2**^**c**^	**0.000**	**< 0.001**	More than 2 mentions associated with lowest HEI score (66 points), no mentions and 1–2 mentions had similar and higher HEI scores (72–73 points).
Decrease alcohol		**0.020**	**0.027**	Mention associated with lower HEI score (63 points) compared to no mention (69 points).
**Increased HEI-C**				
Poultry more than red meat		**0.001**	**0.001**	Mention associated with higher HEI score (76 points) compared to no mention (68 points).
Increase plant protein		**0.000**	**< 0.001**	Mention associated with higher HEI score (73 points) compared to no mention (66 points).
Increase fish		**0.000**	**< 0.001**	Mention associated with higher HEI score (72 points) compared to no mention (66 points).
Eating breakfast		**0.006**	**0.007**	Mention associated with higher HEI score (72 points) compared to no mention (66 points).
Increase milk and alternatives		**0.008**	**0.009**	Mention associated with higher HEI score (71 points) compared to no mention (67 points).
Choosing healthier fats		**0.019**	**0.013**	Mention associated with higher HEI score (71 points) compared to no mention (67 points).
Increase olive oil		**0.022**	**0.036**	Mention associated with higher HEI score (72 points) compared to no mention (68 points).
Increase nuts		**0.036**	**0.030**	Mention associated with higher HEI score (70 points) compared to no mention (67 points).
Increase fruits and Vegetables	**0, 1–2,>2**^**d**^	**0.039**	**0.023**	Any mention associated with higher HEI score (70 points) compared to no mention (65 points).
Healthier snacks		**0.045**	**0.028**	Mention associated with higher HEI score (70 points) compared to no mention (67 points).
**No Difference**				
Increase fibre		0.077	0.053	
Decrease total fat		0.511	0.470	
Decrease sodium		0.263	0.229	
Regular meal pattern		0.357	0.424	
Healthier choices when eating out		0.414	0.414	
Decrease calories		0.691	0.873	
Mindful eating approaches		0.484	0.751	
Decrease intake of added sugars		0.842	0.804	
Decrease glycemic index		0.874	0.828	
Plant sterols	**Not analyzed**			
Increase wine	**Not analyzed**			
Carbohydrate counting	**Not analyzed**			

^a^ Levene’s test of equality of error variance of the dependent variable was p > 0.05 for all univariate analyses

^b^Adjusted for sex, age, BMI, Baseline percentile VO_2_ max, Baseline PROCAM score, spouse yn

^c^Balanced meals recoded as: 0 = not mentioned (*n* = 34); 1 = mentioned 1–2 times in 3 months (*n* = 63); 2 = mentioned more than twice (*n* = 158)

^d^Increased fruits and vegetables recoded as: 0 = not mentioned (*n* = 80); 1 = mentioned 1–2 times in 3 months (*n* = 66); 2 = mentioned more than twice (*n* = 109)

Among the six FBG mentioned most often (at least 5% of the time), *Balanced meals* had an inverse association with 3-month HEI-C, while *Increase fruits and vegetables*, *Increase fish*, *Healthier snacks* and *Increase nuts* were associated with statistically increased HEI-C. *Decrease sodium* and *Regular meal pattern* were not associated with any difference in HEI-C at 3-months. Analysis of *Increase plant sterols* or *Wine, if consuming alcohol* was not done as these goals were used so infrequently.

## Discussion

To make progress in linking personalized diet counselling process for CMR conditions to intermediate outputs, we need to know first what interventions are being offered and then if they are associated with relevant outputs, like short-term changes in diet quality. The literature was searched for any comparable studies and none were found that holistically addressed both the content or food aspects of diet counselling, as well as the behaviour change techniques being used. Within the context of implementation studies, it is also relevant to consider the feasibility and value of data collection methods. A major challenge is to document process in sufficient detail to detect important differences, while remaining feasible in the typical practice setting. Health behaviour counselling is a complex activity and researchers in past studies have been considering care process at many levels, from basic paper review of study methods in published papers to video review of observed clinical encounters. This analysis represents an attempt to use RD self-report as a possible approach to document key aspects of process that impact diet quality.

Use of diet quality as a short-term output is still relatively uncommon, but is increasing, as more researchers are publishing personalized nutrition studies. The value of such tools lies in assessing both baseline diet quality relative to the population as well as assessing change in groups after intervention. For example, the large European Food4Me study (*n* = 1269 completers) tested the hypothesis that subjects given phenotype and genotype data would improve diet quality more in a 6-month internet counselling study, compared to control subjects [71]. The 2010 US HEI was calculated, and all intervention groups combined demonstrated a 4-point mean change over 6 months, compared to a 2.3 point change in the control group, for a between group difference, adjusted for baseline of 1.27 points (95% CI, 0.30, 2.25, *p* = 0.010). Baseline HEI values were 49.1 to 49.5, slightly lower than the mean of 55.4 (SD = 8.32) found in a German household study [72]. The few studies using versions of the HEI in MetS have shown results similar to ours, in terms of increases in fruits and vegetables and reductions in sugar intake as examples [73, 74]. More work on the development and measurement properties of diet quality tools to assess dietary change in counselling studies is warranted and may yield important insights in interpreting the results of different studies.

Documentation of encounter time and channel in diet counselling is common [75], but there is little agreement on best practices for number or types of contact. In Mitchell et al.’s review of clinical trials of dietetic consultations in primary care there was a range of 19 consultations (mean 5.6); and 25–600 minutes of contact time [76]. In our study, the decision to fix the number of encounters was intended to ensure sufficient dose of intervention and potential for clinical indicators to change, for all participants. In considering our results with other diet studies, multiple contacts over time seem to be important for people to practice new skills and to help develop new food habits. Since almost all contacts were in-person in this study, possible differences in effectiveness by channel (email, phone, video) were not considered, and await further work. Most changes in our study had occurred by 3 months. More work is needed to better define ranges for maximal effectiveness in most patients. In this regard, interventions that focus on weight loss vs changes in diet quality should be considered separately, as diets for weight loss involve restriction of caloric intake and increased hunger, suggesting a possible need for different types and length of encounter time, to maximize potential for adherence. Changes in diet quality persisted to 12 months in this study that did not have a strong focus on weight loss. Evidence for longer term maintenance of changes to diet quality are limited.

Multiple FBG were documented by the RDs throughout the study; on average > 2 FBGs/person/contact. There was a strong emphasis on the *Balanced meals* and the ‘Healthy plate’ concept as measured by frequency of mention and use of these patient resources, in line with Canada’s Food Guide in current use during the study [54]. Surprisingly, however, the more times the concept was documented, the more adverse the 3-month HEI-C, compared to when it was mentioned fewer times or not at all. In post-study interviews, a few RDs mentioned starting with the *Balanced meals* concept before moving on to specific food advice but indicated that if subjects did not make progress on eating more balanced meals, the concepts would be reviewed again (Stevens, undergraduate thesis). Further work is needed to unravel cause and effect, as well as the utility of the balanced meals concept, for counselling different patients. It was not surprising that counselling to *decrease alcohol* would be associated with lower 3-month HEI-C, although no specific intervention study on this topic was found.

Goals of consuming *poultry more often than red meats, increasing plant protein, increasing fish and eating breakfast* were less commonly promoted, yet were associated with potentially clinically relevant 6-point higher 3-month HEI-C [74, 77]. From population studies, it is known that the standard deviation of HEI scores tend to be in the range of 12 points (similar in our study); therefore a 6-point difference in HEI-C would represent an effect size of ~ 0.5; which many people would consider a “medium” effect [77].

Looking over the rest of the FBG, it can be noted that goals defined in terms of specific food changes were associated with statistically increased HEI-C in the expected direction, but of lesser magnitude, while FBG that were focused on reducing nutrients (sodium, total fats, calories) or aimed at affecting the whole diet (glycemic index, mindful eating) were not associated with higher HEI-C, compared to not using that FBG. Our null results for mindful eating are consistent with results of a recent meta-analysis [78].

With respect to use of 16 specific BCT, *Goal setting* is an essential task in counselling and accounted for 15% of all BCTs recorded, yet more goal setting (>3times/3 months) was associated with a statistically poorer HEI-C at 3 months (− 5 points), compared to fewer or no mentions, which did not differ from each other. Reasons for more goal setting being associated with poorer HEI-C may have reflected challenges in the joint goal setting process. Goal setting is well documented as an essential BCT; however adverse effects of more goal setting, over multiple visits has not been previously reported to our knowledge, as most reviews to date have only reviewed written descriptions of study methods [79]. Some have suggested that more goal setting may be a symptom of issues with achieving realistic goals within the constraints of daily life, which vary tremendously from person to person. Further work to explain these associations is needed.

More S*elf-monitoring* (> 4 mentions) was associated with improved HEI-C, and the HEI-C of the no mention and 1–4 mention groups did not differ from each other, which is consistent with the literature [35, 80–83] and RD experience [59]. *Focus on past success* was associated with a statistically significant 4-point higher HEI-C at 3 months, as would be expected. *Feedback on past performance,* however, found that HEI-C scores were 4 points higher in the intermediate category, and overall association F test was relatively weak (*p* < 0.037). Further work is needed to see if the two approaches differ in effectiveness. None of the rest of the BCTs were statistically associated with improved HEI-C.

Several meta-analyses have used the CALO-RE taxonomy to complete paper reviews of the BCT used in studies of adults where at least some studies related to dietary change outcomes [84–89]. Results were mixed. Four of the six studies attempted to relate use of BCT to an outcome. Goodwin et al. did not find an association with mortality [84], nor did McDermott et al. find an association with physical activity or eating behaviours [86]. In weight loss studies, Hartman et al. found that “comparing behaviour with others” was the only BCT associated with greater weight loss [87]. We did not record this BCT. Lara et al. found that several BCT were associated with increased intake of fruits and vegetables, specifically “planning social support”, “goal setting”, “follow-up prompts” and “providing feedback on performance” [88]. This was most comparable to our analysis as we used three of these BCT, excluding follow-up prompts as study contacts were fixed. One relevant study used the CALO-RE taxonomy to analyse BCT observed in video-recordings of initial consultations to promote exercise among older adults [90]. “Providing information about where and when to perform the behavior” (86%) and “setting outcome goals” (82%) were used most often. Self-monitoring was rarely mentioned. In this study, a key issue may have been that it was an initial consultation.

More recent relevant results have used the later Behaviour Change Technique Taxonomy version1, BCTTv1 [65], which greatly expanded the number of BCT from 40 in the CALO-RE taxonomy to 93 items. Alageel et al. conducted a relevant review of CVD prevention in primary care [91] that found highly heterogenous results and limited use of BCT. Cradock et al. reviewed diet and physical activity interventions in diabetes for changes in HbA1c and weight [92]. Four of 46 BCT identified were associated with > 0.3% reduction in HbA1c: ‘instruction on how to perform a behaviour’, ‘behavioural practice/rehearsal’, ‘demonstration of the behaviour’ and ‘action planning’ Samdal et al. reviewed physical activity and healthy eating in overweight and obese adults, but focused on assessing BCT used only with intervention groups and not control groups, and combining diet and exercise [83]. Goal-setting and feedback on outcomes of performance were significant in meta-regression in the long-term. Technology driven diabetes prevention studies were reviewed by Van Rhoon et al. [93]. The techniques of social support (unspecified), goal setting (outcome/behaviour), feedback on behaviour, and self-monitoring of outcome(s) of behaviour were identified in over 90% of interventions, effective for weight loss (3% at 6 months; ≥ 5% at 12 months).

Our analysis appears to be among the first to use self-report of BCT by practitioners to document process and to consider changes in BCT over the course of an intervention. A benefit of having practitioners document their BCT is that we are likely to be obtain more accurate and detailed information compared to a review of study descriptions as reported in published papers. A key challenge, however, is feasibility. We were aware of this from the outset and completed RD reviews to limit the number of BCT, used a training video and created drop-down menus for quicker data entry. The finding that only 2.0 BCT/person/contact to 3-months were documented, suggests substantial under-reporting was occurring. BCT have been defined as the smallest identifiable components that in themselves have the potential to change behaviour [66]. Clearly, in any counselling or group program many BCT are being used. For example, MacPherson et al. used the BCT taxonomy v1 to document BCT they used in group classes for diabetes prevention and identified 30 BCT per session [94]. Therefore, the BCT reported by the RDs were their perceptions of the important or noteworthy BCT in the session. It may be worthwhile in future work to only focus on a few BCT if self-report methods will be used. For example, we found two older analyses that were relevant. One study was found of coaching methods used in the Diabetes Prevention Program study, where coaches documented their coaching techniques [95]. Lifestyle coaches used problem-solving with most participants and regularly reviewed self-monitoring skills. In the other study, participants in a long-term clinical trial (ADDITION-Plus) reported on their own use of eight BCT related to diet and physical activity at 1 year. “Goal setting”, “goal review” and “preparing for setbacks” was related to decreased % fat in diet, but not to physical activity [96]. Given our experience in the challenges RDs had in documenting BCT and the current very long lists of BCT that have been developed, there is a need to find some middle ground for practical assessment of groups of BCT that can be used in future effectiveness studies of diet counselling [66]. Further methodological work is needed to identify the BCT that capture differences in techniques that are feasible to measure and can be associated with outputs.

RDs are often educating on food skills, and this is explicit in the NCP terminology [75]. This focus is well justified by evidence of poor food skills among many Canadians [97]. The CALO-RE taxonomy was not very explicit on this aspect, as also noted by Hollywood et al. in their review of food skills interventions [85]. The RDs in this study made variable use of the option to add food skills to their documentation. We believe skills training was under-reported in this study and needs to be addressed in future studies. The more recent version of the 93 item Behavioural Change Taxonomy v.1 has addressed this gap, through 2 of 19 categories (‘Shaping Knowledge’ and ‘Repetition and Substitution’) [65].

Other work in health behaviour change may have application to future diet counselling studies. There has been increasing emphasis on the concept of fidelity in delivery of complex behavioural interventions, with use of video recording and analysis to document process and content. Two examples for RD counselling in cancer patients [98] and weight loss [99] were located, but they did not use the BCT taxonomies to describe process. Given our state of knowledge, we believe a strong focus on fidelity may be premature within personalized diet counselling. For example, use of motivational interviewing has been widely promoted in the profession and was very commonly reported in this study (10% of all BCTs), yet there was no association with change in diet quality. We believe a more directed approach is now needed to identify the key BCT, FBG and skills training that can be explicitly associated with relevant diet change.

Participant engagement, the degree that participants will use tools from the intervention in their lives, is a final area requiring more work. While we conducted an overall patient satisfaction study as part of the CHANGE project [44], and asked about confidence to maintain diet change, we did not assess patient engagement in detail. Walton et al. [100] completed an interesting systematic review of 66 studies for complex health interventions assessing both fidelity and engagement, only 6 of which included a diet intervention. Members of the group have published a five-step process for developing engagement measures for implementation studies that may be useful [101].

### Strengths and limitations

This is one of the first studies to attempt to link dietetic care process assessed by practitioners themselves to short-term counselling outcomes. We have succeeded in demonstrating significant associations for several FBG to changes in diet quality in a well conducted pre-post study. We were less successful in eliciting BCT. The self-report format was feasible for a primary care-based study, and efforts were made to ensure reliable documentation of counselling over the 4-year study. Use of a generic diet quality measure, the HEI-C, was a strength in that it was possible to quantify several promoted food intake changes in a single measure.

A self-report format using a check-all-that-apply list of possible FBG and BCT was employed as is common in clinical studies, but the wide variability in overall reporting suggests that RDs varied in the extent of recording. Further work to assess reliability and validity of self-report methods is now justified. For example, it is known that forced choice responses will elicit more responses, but both methods result in similar rank ordering [102]. Our analysis approach was very basic, being limited to descriptive statistics and univariate associations and ignored the complexity and likely interactions among different elements of the counselling process.

## Conclusions

Our approach was unique in building on typical RD practice and not imposing a particular “diet” as is common in most clinical nutrition research. While RDs promoted certain eating patterns such as Canada’s Food Guide and the Mediterranean diet, they were encouraged to use their professional judgment and skills in personalizing to the needs of patients and had the opportunity to provide a far more intensive intervention than is typical in the health care system. Assessing process of care under these ideal conditions, has allowed us to begin to open the “black box” of dietetic counselling, in line with work by others in the health behaviour change field to identify key aspects that impact diet quality. Further work s needed to demonstrate the links between counselling and outcomes, and also improve overall effectiveness of personalized diet counselling. We believe that adapting the NCP terminology with the most current behaviour change taxonomy from Michie et al. group [65, 66] to assess effects of multiple process factors on short-term (3 month) food intake changes, using simplified scores such as diet quality or other measures, will be a fruitful strategy going forward.

## Supplementary Information

**Additional file 1.** Behavioural Change Strategies used in Counselling. (Adapted from Michie et al., 2011)^1^.

**Additional file 2.** Food Behaviour Goals Used in Counselling.

**Additional file 3.** Components, range of scores, and scoring criteria for Canadian Healthy Eating Index (HEI-C).

## Data Availability

The datasets used and/or analysed during the current study are available from the corresponding author on reasonable request.
