# Supplementary material for: Key process features of personalized diet counselling in metabolic syndrome: secondary analysis of feasibility study in primary care
Source: BMC Nutr. 2022 May 9;8:45. doi: 10.1186/s40795-022-00540-9 (PMC9081667; doi:10.1186/s40795-022-00540-9)
Supplement: Supplementary file 1 — Additional file 1. Behavioural Change Strategies used in Counselling. (Adapted from Michie et al., 2011)1. [file 40795_2022_540_MOESM1_ESM.docx]

**Additional File 1**. Behavioural Change Strategies used in Counselling (Adapted from Michie et al., 2011)^1^

Based on the descriptions provided below, dietitians were instructed to check top 4 behavioural change strategies that apply to the intervention for each encounter.

|  | **Description** | **Used in CHANGE project**^2^ |
| --- | --- | --- |
| 1. Consequences of behaviour in general, provide information on | Provide information on relationship between behaviour and general consequences based on epidemiological data. | **X**  (combined together as ‘consequences of behaviour’) |
| 1. Consequences of behaviour to the individual, provide information on | Provide information on the benefits and costs of action or inaction to the individual or group. |  |
| 1. Others' approval, provide information about | Provide information about what other’s think of client’s behaviour (including approval and disapproval). |  |
| 1. Other's behaviour, provide normative information about | Provide information about what other people are doing amongst a population or specified group.  **Note:** Different than strategy 3 as it focuses on actions not approval |  |
| 1. Goal setting (behaviour) | Encouraging the client to make a behavioural resolution.  **Note:** Different than strategy 6 or 7 as it does not involve planning exactly how the behaviour will be executed or when/where the behaviour will be performed. | **X** |
| 1. Goal setting (outcome) | Encouraging the client to set a general goal that is be achieved by behavioural means but is not defined in terms of behaviour. |  |
| 1. Action planning | Detailed planning of what the person will do when, in which situation and/or where to act. ‘When’ can describe frequency or duration. | **X** |
| 1. Barrier identification/problem solving | After setting a goal, clients are asked to identify barriers to achieving this goal  **Note:** Differs from 7 and 9 because it focuses on obstacles to performance. | **X** |
| 1. Graded tasks, set | Breaking down goal behaviour into smaller tasks that incrementally increase in difficulty over time  **Note:** Differs from strategy 7 which focuses more on "when/where" contingencies to perform behaviours. | **X** |
| 1. Review of behavioural goals, prompt | Review extent to which previously set behavioural goals were achieved, followed by a revision or goals and means to attain them, if necessary. | **X** |
| 1. Review of outcome goals, prompt | Review of the extent to which previously set outcome goals were achieved followed by a revision or goals and means to attain them, if necessary. |  |
| 1. Rewards contingent on effort or progress towards behaviour, prompt | Using praise or rewards to attempts at a behavioural goal (includes self-reward).  **Note:** Differs from technique 13 which is reinforcement of performing target behaviour itself. | **X** |
| 1. Rewards contingent on successful behaviour, provide | Reinforcing successful performance of specific target behaviours. Includes praise, encouragement, material rewards and self-reward.  **Note:** Different than strategy 7, 17 and 19 |  |
| 1. Shaping | Graded use of contingent rewards over time. Rewards are first provided for an approximation of the target behaviour (any behaviour change) and then later, only more demanding performance is rewarded. This includes praise, encouragement, material rewards and self-reward. |  |
| 1. Generalization of a target behaviour, prompting | Once behaviour is performed in a particular situation, the person is encouraged or helped to try it in another situation. |  |
| 1. Self-monitoring of behaviour, prompt | Person is asked to keep a record of specified behaviour as a method of changing behaviour.  **Note:** Differs from strategy 17 which focuses on tracking outcomes. | **X** |
| 1. Self-monitoring of behaviour outcome, prompt | Client is asked to keep a record of specified measures expected to be influenced by behaviour change. |  |
| 1. Focus on past success, prompt | Instructing the client to think about or list previous successes in performing the behaviour (or parts of it). Not just encouragement or feedback. | **X** |
| 1. Feedback on performance, provide | Providing the client with data about their own recorded behaviour (following strategy 16) or commenting on person's behavioural performance (following strategy 5 or 7) or discrepancy between client's own performance in relation to others (likely also involves strategy 28). | **X** |
| 1. Information on where and when to perform the behaviour, provide | Providing information on when and where a client may be able to perform the behaviour. |  |
| 1. Instruction on how to perform the behaviour, provide | Telling the client how to perform behaviour or preparatory behaviours, either verbally or in written form.  **Note:** Differs from showing how to perform - see strategy 22 |  |
| 1. Model/demonstrate the behaviour | Showing the person how to perform a behaviour through physical or visual demonstrations. |  |
| 1. Cues/prompts, teach to use | Teach client to identify environmental cues that can be used to remind client to perform certain behaviour. | **X** |
| 1. Environmental restructuring | Client is prompted to alter the environment in ways so that it is more supportive of the target behaviour. | **X** |
| 1. Behavioural contract, agree to a | A (verbal or) written agreement outlining performance on an explicit behaviour. | **X** |
| 1. Practice, prompt | Ask client to rehearse and repeat behaviour either in the session or between sessions. **AKA** building habits or routines. |  |
| 1. Follow-up prompts, use of | Contact with the client outside this session meant to remind them to complete a task or work on a behaviour. |  |
| 1. Social comparison, facilitate | Explicitly drawing attention to other’s performance to elicit comparisons (takes place in group settings). |  |
| 1. Social support/social change, plan | Prompting the client to plan how to elicit social support from other people to help him/her achieve their target outcome / behaviour. | **X** |
| 1. Role model/position advocate, prompt identification as | Focusing on how the client can be a role model to others (such as children). OR Providing opportunities for the client to persuade others of the importance of adopting the behaviour. |  |
| 1. Anticipated regret, prompt | Inducing expectations of future regrets about the performance or non-performance of a behaviour. Focus on feelings in the future.  **Note:** Different than strategy 1 and 2 as they provide more generic information |  |
| 1. Fear arousal | Presentation of risk and/or mortality information relevant to the behaviour as emotive images designed to evoke a fearful response. |  |
| 1. Self talk, prompt | Encouraging client to talk to themselves (aloud or silently) during or before the goal behaviour to encourage, support and maintain action. |  |
| 1. Imagery, prompt use of | Teach the person to imagine successfully performing the behaviour or to imagine finding it easy to perform the behaviour.  **Note:** Different that asking client to recall instances of past success (strategy 18) |  |
| 1. Relapse prevention/coping strategies | Creating a plan on how to maintain a new behaviour by creating strategies to avoid/cope with relapse situations.  **Note**: Contrasts with strategy 7 and 8 which are about initiating behaviour change | **X** |
| 1. Stress management/emotional control training | Using a specific set of techniques which do not target the behaviour directly but seek to reduce anxiety and stress to facilitate the performance of the behaviour.  **Note:** May be paired with strategy 8 by identifying emotional barriers |  |
| 1. Motivational interviewing | Clinical counselling method aimed at prompting the client to engage in change talk in order to minimize resistance and resolve ambivalence to change. | **X** |
| 1. Time management | Use of any technique designed to teach a person who to manage their time in order to make time for the behaviour. |  |
| 1. General communication skills training* | Using any technique directed at general communication skills but not towards a specific behaviour change. |  |
| 1. Anticipation of future rewards, stimulate | Creating anticipation of future rewards without necessarily reinforcing behaviour through the active period of intervention. |  |
| 1. other |  | **X**  (specify) |

^1^ Michie S, Ashford S, Sniehotta FF, Dombrowski SU, Bishop A, French DP. A refined taxonomy of behaviour change techniques to help people change their physical activity and healthy eating behaviours: the CALO-RE taxonomy. Psychol Health. 2011;26(11):1479-98.

^2^ CHANGE, Canadian Health Advanced by Nutrition & Graded Exercise project
