# Supplementary material for: Key process features of personalized diet counselling in metabolic syndrome: secondary analysis of feasibility study in primary care
Source: BMC Nutr. 2022 May 9;8:45. doi: 10.1186/s40795-022-00540-9 (PMC9081667; doi:10.1186/s40795-022-00540-9)
Supplement: Supplementary file 2 — Additional file 2. Food Behaviour Goals Used in Counselling. [file 40795_2022_540_MOESM2_ESM.docx]

**Additional File 2.** Food Behaviour Goals Used in Counselling

From the list below, for each encounter (contact with patient) dietitians were instructed to record all food behaviour goals that were addressed, indicate which stage the patient is on (i.e. whether the goal was ‘set’, ‘being worked on’ or ‘attained’) and to record up to 3 resources used to support the goal.

| **Food Behaviour Goal** | **Stage**  **‘**set’, ‘being worked on’ or ‘attained’ | **Resources Used**  (up to 3 resources) |
| --- | --- | --- |
| None | -- | -- |
| Decrease intake added sugars |  |  |
| Balanced meals |  |  |
| Regular meal pattern |  |  |
| Eating breakfast |  |  |
| Carbohydrate counting |  |  |
| Healthier choices when eating out |  |  |
| Decrease calories |  |  |
| Choosing healthier fats |  |  |
| Increase olive oil |  |  |
| Decrease total fat |  |  |
| Increase fibre |  |  |
| Decrease glycemic index |  |  |
| Increase nuts |  |  |
| Increase plant protein |  |  |
| Increase plant sterols |  |  |
| Increase fish |  |  |
| Poultry more than red meat |  |  |
| Increase milk and alternatives |  |  |
| Healthier snacks |  |  |
| Decrease sodium |  |  |
| Increase fruits / vegetables |  |  |
| Wine if consuming alcohol |  |  |
| Decrease alcohol |  |  |
| Mindful eating approaches |  |  |
| Other (specify) |  |  |
