# Supplementary material for: Key process features of personalized diet counselling in metabolic syndrome: secondary analysis of feasibility study in primary care
Source: BMC Nutr. 2022 May 9;8:45. doi: 10.1186/s40795-022-00540-9 (PMC9081667; doi:10.1186/s40795-022-00540-9)
Supplement: Supplementary file 3 — Additional file 3. Components, range of scores, and scoring criteria for Canadian Healthy Eating Index (HEI-C). [file 40795_2022_540_MOESM3_ESM.docx]

**Additional File 3.** Components, range of scores, and scoring criteria for Canadian Healthy Eating Index (HEI-C).

| **Component** | **Range of scores** | **Scoring criteria*** |
| --- | --- | --- |
| ***Adequacy**** | **0 to 60** |  |
| Total vegetables and fruit | 0 to 10 | Maximum: 4 to 10 servings^1,2^ |
| Whole fruit | 0 to 5 | Maximum: 0.8 to 2.1 servings^1-3^ |
| Dark green and orange vegetables | 0 to 5 | Maximum: 0.8 to 2.1 servings^1-3^ |
| Total grain products | 0 to 5 | Maximum: 3 to 8 servings^1,2^ |
| Whole grains | 0 to 5 | Maximum: 1.5 to 4 servings^1,2,4^ |
| Milk and alternatives | 0 to 10 | Maximum: 2 to 4 servings^1,2^ |
| Meat and alternatives | 0 to 10 | Maximum: 1 to 3 servings (75 to 225grams)^1,2^ |
| Unsaturated fats | 0 to 10 | Maximum: 30 to 45 grams^1,2^ |
| ***Moderation**** | **0 to 40** |  |
| Saturated fats | 8 to10 | 7% (score of 10) to 10% (score of 8) of total energy |
|  | 0 to 8 | 10% (score of 8) to 15% (score of 0) of total energy |
| Sodium | 8 to 10 | Adequate intake (score of 10) to tolerable upper intake level (score of 8) |
|  | 0 to 8 | Tolerable upper intake level (score of 8) to twice tolerable (score of 0) |
| “Other food” | 0 to 20 | Minimum: 40% or more of total energy intake  Maximum: 5% or less of total energy intake |
| ^*^Proportional scores are given for amounts between the minimum and maximum  ^1^Age and gender specific recommendations from Canada’s Food Guide (2007)  ^2^Minimum of 0 serving  ^3^21% of recommendation for total vegetables and fruit  ^4^50% of recommendation for total grain products | | |
